# "Did the trial kill the intervention?" experiences from the development, implementation and evaluation of a complex intervention

**DOI:** 10.1186/1471-2288-11-24

**Published:** 2011-03-01

**Authors:** Lydia Bird, Antony Arthur, Karen Cox

**Affiliations:** 1 Faculty of Medicine and Health Sciences, School of Nursing, Midwifery and Physiotherapy, B49 Research Office, Queen's Medical Centre, University of Nottingham, NG7 2HA, UK

## Abstract

**Background:**

The development, implementation and evaluation of any new health intervention is complex. This paper uses experiences from the design, implementation and evaluation of a rehabilitation programme to shed light on, and prompt discussion around, some of the complexities involved in such an undertaking.

**Methods:**

Semi-structured interviews were conducted with 15 trial participants and five members of staff at the conclusion of a trial evaluating a rehabilitation programme aimed at promoting recovery after stem cell transplantation.

**Results:**

This study identified a number of challenges relating to the development and evaluation of complex interventions. The difficulty of providing a standardised intervention that was acceptable to patients was highlighted in the participant interviews. Trial participants and some members of staff found the concept of equipoise and randomisation challenging and there was discord between the psychosocial nature of the intervention and the predominant bio-medical culture in which the research took place.

**Conclusions:**

A lack of scientific evidence as to the efficacy of an intervention does not preclude staff and patients holding strong views about the benefits of an intervention. The evaluation of complex interventions should, where possible, facilitate not restrict that complexity. Within the local environment where the trial is conducted, acquiescence from those in positions of authority is insufficient; commitment to the trial is required.

## Background

The development of the randomised controlled trial has radically altered the way in which medical therapies are developed, tested and administered. Since 1947 when the Medical Research Council initiated what is generally considered to be the first randomised and blinded clinical trial [1,2] the principles of randomisation and control have moved from being controversial novelties to expected normalities. In the 1990's the broadening of the concept of evidence based medicine towards evidence based practice reflected a growing recognition of the need for decisions about health care interventions to be based on evidence of effectiveness.

However, there are obvious differences between the evaluation of a new drug and, for example, the evaluation of an intervention to promote recovery after cancer treatment and it is not always possible to simply extend the randomised controlled trial design. In acknowledgment of this the Medical Research Council developed in 2000 [3] and then revised in 2008 [4] guidance for the development and evaluation of complex interventions. The MRC emphasise the need for robust and rigorous evaluation of complex interventions, promoting the use of experimental methods, but providing information on some of the alternatives to the conventional randomised controlled trial and highlighting situations in which a trial is impractical or undesirable [4].

Given the current financial imperative for interventions to be of proven benefit in order to compete for finite resources, the focus on patient centred care and the undisputed value of the randomised controlled trial it is likely that the number of trials of complex interventions will increase considerably. With this in mind we wanted to provide comment on one randomised controlled trial of a complex intervention which was recently conducted in order to explore some of the acknowledged and hidden complexities of this form of research.

This paper reports findings from a qualitative study of the experiences of the development, implementation and evaluation of a rehabilitation programme following stem cell transplantation in a regional haematology unit. A number of staff working on the unit had identified a need for more structured rehabilitation that might include not only support for patients' physical problems but also would address some of the perceived social and psychological needs of these patients. A programme of rehabilitation based on evidence from both the cancer and cardiac rehabilitation literature (for example [5-7]) was put together by a small group of nursing and physiotherapy staff working in collaboration with the rest of the clinical team and patients who had previously undergone stem cell transplant. The programme was piloted by these staff members who felt it was a viable model of routine service delivery and observed positive effects among the small number of patients who undertook the pilot programme. Since these results were based on a small, uncontrolled sample and conducted by those who had developed the intervention, the possibility of bias is a legitimate concern. At this stage an external research team was appointed to conduct an independent and definitive study that attempted to answer whether or not the programme was effective in improving patient outcomes. This paper aims to shed light on, and prompt discussion around, some of the complexities involved in undertaking a randomised controlled trial of two forms of rehabilitation (healthcare professional led and self-managed).

## Methods

The design of the evaluation was mixed-methods with a qualitative interview study following the completion of the randomised controlled trial. A full description of the trial and the quantitative results are reported elsewhere [8] but brief details are provided here to give context to the qualitative study that is the focus of this paper.

The trial design chosen was a two-arm parallel study comparing structured rehabilitation in a hospital setting led by a team of health professionals (HPL programme) with a home-based, self-managed rehabilitation programme (SM programme). Participants were aged 18 years and over, and had been treated in the previous six to eight weeks with an autologous or allogenic stem cell transplant. The primary outcome was change in physical functioning at six months. Potential participants were initially approached by a healthcare professional and then contacted by a member of the research team (LB) and at this point if potential participants were happy to take part they were asked to provide formal written consent. Following consent and baseline data collection, LB informed participants of their allocation after telephoning an individual independent to the study team who held the randomisation lists.

Participants randomised to the HPL programme were asked to attend the hospital once a week for 10 weeks to take part in a group session consisting of a tailored exercise programme, and a relaxation and information support session. Participants randomised to the SM programme were given an information pack that contained a home-based exercise programme and access to all the information and relaxation exercises provided on the HPL programme, this being a slight enhancement of the standard care where rehabilitation was provided in a less structured and more ad hoc manner. Patients could not access the intervention outside of the trial.

During a 14-month recruitment period 144 potential participants were approached about the trial and 61 (42%) consented to take part. Common reasons for declining to take part were distance from home to hospital, and that the programme did not seem relevant. Fifty-eight people were randomised and 46 participants were followed-up at six months. The main reasons for drop out were disease relapse and death.

## Qualitative interviews

Individual qualitative interviews to gather the rehabilitation and trial experiences of those who took part in the trial were included in the original study design. The study design was revised to incorporate further individual interviews with staff members in order to explore their perspectives on the challenges that arose during the trial. The interviews were all conducted by the same researcher (LB) as had managed the trial. The researcher had a background in nursing but did not have any clinical role on the unit where the trial took place. The study was approved by the North Nottinghamshire Local Research Ethics Comittee (LREC reference number 05/Q2402/51).

### Patient interviews

Interviews were conducted with 15 patients from within the sample of 58 participants who took part in the trial (see table 1). Participants were interviewed shortly after the end of their involvement in the trial and interviews explored patients' experiences of rehabilitation and of participation in a randomised controlled trial.

**Table 1:** Characteristics of interviewees

	Intervention (n = 7)	Control (n = 8)	Total
**Participant Interviewees (n = 15)**			
**Gender**			
Male	2	5	7
Female	5	3	8
**Age**			
< = 50	3	4	7
> 50	4	4	8
**Transplant type**			
Allograft	3	2	5
Autologous	4	6	10
**Staff Interviewees (n = 5)**			
**Occupation**			
Physiotherapist			1
Nurse			2
Doctor			2
**Direct involvement in the intervention**			
Yes			3
No			2

Maximum variation sampling was used to ensure that the sample included women and men, individuals from both arms of the trial, and across age groups. All participants approached to be interviewed agreed to participate. The interviews lasted between one to one and a half hours and were conducted at a location convenient for the participant. This tended to be in a quiet side room at the hospital or in the participant's own home. Topic guides covered life after transplantation, experiences of rehabilitation, the acceptability of randomisation, the acceptability of the evaluation tools and overall trial experience. The schedules served as a guide for the interview structure and content but did not stipulate exact phrasing of questions and prompts, or sequence of areas of enquiry.

### Staff interviews

Interviews were also conducted with five members of staff, see table 1, after completion of the trial. All three staff members who had been instrumental in designing and implementing the health profession led programme were interviewed. Two other individuals were also interviewed and whilst they had no direct involvement in the design or delivery of the healthcare professional led programme, it was felt that these individuals were responsible for setting the priorities and ethos of the transplant unit. All staff members approached to be interviewed agreed to participate. The staff interviews explored attitudes and beliefs about the rehabilitation programme and about the appropriateness of evaluating it using a randomised controlled trial. Interviews with members of staff who had been involved in running the rehabilitation programme explored the experience of providing the rehabilitation programme within the context of a randomised controlled trial. The interviews were conducted during the working day in a location convenient for the staff member, typically their own office or a private meeting room. Topic guides were used to provide a structure for the interviews.

### Analysis

The analysis of the qualitative data was guided by the need to have a more contextual and enhanced [9] understanding of the trial. All interviews were audio recorded, transcribed verbatim and then analysed using NVivo 8. A thematic content approach to data analysis was used. Following transcription, the interview transcripts were checked for accuracy, read for context and then coded. Individual codes were then collapsed into broader categories that could be generally divided between the two main areas of enquiry (rehabilitation and evaluation) that were the focus of this qualitative study. Within these two areas, themes could be distinguished that helped to explain both commonalities and differences in patient and staff perspectives. The analysis was conducted by two of the authors (LB and AA) and corroborated by the third (KC). In the findings section quotes from trial participants are identified with a P followed by a study number and then the abbreviation HPL denoting that they were allocated to the healthcare professional led programme or SM denoting that they were allocated to the self-managed programme. Quotes from staff are identified with an S followed by a study number.

## Results

### Patients' perceptions of the rehabilitation programmes

Despite being developed by staff in collaboration with patients and having undergone a pilot phase to test feasibility and acceptability, views of the healthcare professional led rehabilitation programme were mixed. Most of those interviewed who had taken part in the HPL programme were positive about the exercise component of the programme. Many expressed some degree of exhaustion after exercising, but this was temporary and relieved by resting. However two participants reported finding the exercises too exhausting and one commented:

*P123 (HPL) I personally felt that it had perhaps done me more harm than good. And I perhaps thought it was a bit early because I wasn't coping with it very well*.

It has been argued that bone marrow transplantation is one of the most stressful treatments in cancer care [10]. It was for this reason that a relaxation component was included as part of the HPL programme. However this was not a need perceived by all participants.

*P117(SM) I tend to just take every day as it comes... I've gone past that stage of worrying about things*.

Some participants clearly felt uncomfortable with the relaxation sessions and one suggested that taking part was like being "*subjected to sort of séances*" (P123HPL). This participant who had not found the relaxation sessions helpful said "*I think a couple of [group members] were nodding off. I thought for god's sake try and keep awake*" (P123) indicating that, for this participant, falling asleep would have been undesirable and unacceptable. However, in contrast another participant commented that "*I love doing that kind of thing, I could kind of fall asleep*" (P326). While the relaxation component was not universally popular some participants indicated that relaxation sessions had been useful and requested assistance with repeating the exercises at home. These were often the participants who, prior to the intervention, had been noticeably anxious or who had found the transplant process to be traumatic.

In the interviews participants were given the opportunity to comment on each of the information sessions provided as part of the HPL programme. Participants' reflections on the sessions were generally positive but suggested that information was sometimes repetitive, simply stating common sense, or given too late in the transplant process.

*P315 (HPL) But a lot of it was repetition and there was a bit of déjà vu. I'd heard and seen a lot of it before*.

The participants felt that recovery after stem cell transplantation was a highly individual process. One example of this was the reaction of different participants to one of the information components which was led by a hospital chaplain which looked at life after transplant. Participants' feelings about the session varied considerably.

*P326 (HPL): the guy who came from the church he was quite interesting, talking about death and how people feel about dying and how people think, that was interesting. I can remember others but I think he was the most interesting one*.

*P320 (HPL): We had the vicar and he was very nice, but for me he was a bit intrusive and that was no fault of his that was just you know the way that I felt about it*.

Several participants allocated to the self managed programme said that they had never attempted the exercises while others indicated that they gave the exercises a go but quickly lost interest. Reasons for this included a lack of motivation to excercise, or a preference for other activities such as gardening or walking the dog. Those participants who reported not completing the exercises said that they believed that their motivation would have been increased if they had been allocated to the HPL programme. Participants attributed this to two factors: the support provided by a group environment and a feeling of greater confidence exercising where there were professionals available to prevent injury. In contrast to this three participants suggested that they found the programme appropriate and helpful and that they had consistently completed the programme several times a week for a period of many weeks. The differing reactions of individuals to the exercises appeared to relate to several factors. The exercises were completed by those with a high level of motivation to return to a previous state of fitness and by those who had previous direct (for example army training) or indirect (for example a close relative with experience of circuit training) experience of exercise training.

*P311(SM) I found them very good... I first looked at them and thought this looks too easy, but, the situation I was in at the time when I came out of hospital, nothing was easy so they were very good overall. The balance was excellent*.

Between and within the two study groups, opinions on the value of the intervention received were strongly divided. This illustrates some of the difficulties inherent in trying to develop rehabilitation interventions of this sort. For many participants, initial enthusiasm was occasionally followed by a need for greater flexibility. This was particularly a problem for the 'group' nature of the health profession led programme, which, by definition was designed for a 'typical' recovery trajectory that did not always suit individuals with varying needs that fluctuated over time. Conversely, for those allocated to the self-managed care group, a lack of contact with others in the early stages of their rehabilitation meant those with less personal support may have struggled to motivate themselves.

### Staff and patient perceptions of involvement in a trial

The understanding of the rationale for conducting a randomised controlled trial varied between the staff members interviewed. Some perceived a robust evaluation to be the right thing to do in a climate of limited financial resources. Others implied that they were involved not because they wanted to find out if the intervention worked but because they wanted to prove that it worked. This is a subtle but important difference in emphasis which had a number of implications on how staff felt about the trial.

*S4 It [the trial] was the right thing to try to do, yes, I think that's right because clearly it [the intervention] involves expense, and the question is how much value it had*.

*S3: we've had to go through that process [the RCT] and I now appreciate that. Before I resented it [the need to test a new intervention]*.

Such a belief in the inherent value of the healthcare professional led programme led these staff to experience disappointment over the outcome of the randomisation process in particular cases. Staff involved in delivering the HPL programme reported finding it very difficult when participants that they perceived would particularly benefit from the intervention were allocated to the self-managed programme.

*S1 there were certain patients that we saw that, you know, you were desperate for them to get the hospital led programme*.

The impact of this was that some members of staff found both the trial experience and recruiting patients burdensome and attributed problems with delivering the HPL programme to the fact that a trial was being conducted. One member of staff perceived the trial to place a series of hurdles that patients had to overcome (such as consent to trial participation and randomisation) to access the HPL programme. As a result, disappointing trial recruitment meant that fewer patients than anticipated were participating in the HPL at any one time, meaning a small group size changed the very nature of the programme; the staff member suggesting that *"the trial had killed the intervention" *(S2).

A number of participants also held strong opinions with regards to the two programmes. Several participants expressed their conviction that the HPL programme was superior.

*P315 (HPL): I was on the right arm of the trial. It did things better for me*.

*P318 (SM): It was fair as you did, you know, being picked out I suppose it is fair. It's just that I were picked out for the wrong one*.

However not all participants felt that the HPL programme was superior. One participant was allocated the HPL programme but chose never to attend commenting afterwards that they should have consented to take part on the condition that they were allocated to the SM programme.

The comments of both staff and patients highlight that the rigour of trial design can easily cause confusion and or dissatisfaction. That a member of staff commented that 'the trial killed the intervention' is particularly interesting. Their perspective was that since the pilot phase had in their opinion been a success it must have been as a result of the trial that the difficulties arose. However it could be argued that the relatively low recruitment and the level of trial attrition were in fact related primarily to the intervention rather than the trial. More robust piloting of both the intervention and of the recruitment and randomisation process may have identified more accurately where the problems lay.

### The Impact of Organisational Culture

It was consistently recognised by staff that due to the nature of the conditions treated on the unit where the research took place, a highly 'technical' culture existed. It was suggested that this narrow focus on the biological needs of patients resulted in an undervaluing of the psychosocial aspects of health.

S5: "Some of the medics are focused on getting people in and getting people out, and therefore spending half an hour talking to somebody about psychological issues, um, isn't high on their cards."

*S4: "Well there probably is [a need for emotional rehabilitation] although I don't know that we cater for that"*.

This, it was argued, had important consequences for how the service was delivered, the implementation of the intervention, and the success of the trial. It was suggested that this technical perspective somehow 'rubbed off' on patients resulting in them experiencing a narrowing of their own concept of health.

*S2: "And I think they (patients) are probably focusing very much on blood counts and whether they've got GVHD [Graft Versus Host Disease] and if they're doing all right, and actually rehab is like one extra thing to them, it's like, almost like they've closed down the shutters, they can't take on any more, and they're like, no actually I'm fine, I'll be all right, I'll sort it out, and it'll be okay"*.

It was felt that despite the fact that the HPL programme was developed in response to the lack of emphasis on psychosocial care, the programme and its evaluation were potentially undermined by the reluctance of some staff to prioritise psychosocial care. Since some members of the medical team did not place a high priority on psychosocial support they likewise did not prioritise a trial which was attempting to evaluate a biopsychosocial intervention. This issue was a particular problem since it was felt that patients were more likely to take part in the trial if it was mentioned to them by a member of medical staff. Without the support of the whole medical team there was not the momentum to promote and maintain trial recruitment. Furthermore, for one member of staff, the trial put into sharp focus the dynamics of the professional hierarchy that was embedded in the local organisational culture.

S3: "these patients, everything they do, from, even sometimes getting out of bed, they will say well we'll do it if the doctors say we'll do it, because everything's so medically controlled, (...) patients do what the doctors say..."

S2: "I've learned a lot, in terms of the sort of power relations and how to get things done or not done. And I would have thought before the trial, I was quite influential, and actually, when it actually comes to it, you realise you're not that influential at all really."

## Discussion

This qualitative interview study has provided an insight into both the way the two rehabilitation programmes were experienced and the reality of conducting a randomised controlled trial in a health service setting. Although there are other good examples of this [11,12] in the main, trial reports of the context of the intervention(s) and the evaluation are limited. The artificiality of the experimental process has been highlighted [13].

The data in this study highlighted the numerous practical difficulties involved in trying to develop and evaluate a complex intervention and that compromise is often required between the optimum research design and the practicalities of delivering health care in the real world. The patient data in this study illustrated how difficult it is to develop and standardise a complex rehabilitation intervention so that it is acceptable to patients with different needs and preferences. Hawe et al. [14] suggest that too often a complex intervention is reduced to its constituent parts in order for it to fulfil the strict requirements of a randomised controlled trial. In effect this results in a complex intervention being reduced to a series of simple interventions and in doing so fails to acknowledge that a complex intervention has the potential to be more than the sum of its parts [14]. Hawe et al. suggest that inconclusive trials could be avoided if standardisation of the function of an intervention rather than of its form was more widely utilised. They suggest that this would allow for context level adaption and enable tailoring of the intervention to the local environment, which would potentially improve efficacy. In our own study, a trial could have evaluated whether having access to a patient centred rehabilitation service improved patient outcomes as opposed to testing whether a defined set of rehabilitation interventions improved patient outcomes. The service could have included a core set of interventions which were selected and delivered in response to individual patient need.

This study found that some participants and staff felt a sense of misgiving over the use of a randomised controlled trial design to evaluate the rehabilitation programmes. Many patients and staff had clear preferences and this meant that the concepts of equipoise and randomisation were contentious. The fact that patients and members of the general public find the concept of randomisation and equipoise perplexing is widely acknowledged [15-17] and advice exists [18] which aims to assist in explaining this concept to potential trial participants. Whilst the benefit of the programme was unproven, some staff held strong personal beliefs about its efficacy. It has been suggested that only 25% of medical staff can envisage themselves being in personal equipoise and only 18% thought that their patients could be in this state [19]. This raises a number of practical and ethical issues. Collective equipoise may be the justification for randomisation but staff who hold strong views on the likely superiority of the effectiveness of one treatment will have difficulty with seeing patients randomised, while potential participants with clear preferences are unlikely to agree to randomisation. Although this is a potential problem in all trials, it is often magnified in the evaluation of complex interventions due to the necessary drive and determination of individuals to bring about their development. It is unrealistic to expect staff to take a neutral position in relation to something that they have been instrumental in creating.

Analysis of this qualitative data has highlighted that difficulties in conducting the trial stemmed not just from the challenges associated with the complexity of the intervention but also from the complexity of the local organisational culture. The importance of trial context has been acknowledged particularly in relation to complex interventions [3,20]. The impact that context and culture can have on a trial has been highlighted in seminal sociological works by Oakley [21] and Fox [22] though trialists have perhaps failed to embrace this literature in a way that can fundamentally change the approach taken to designing and implementing clinical trials. Each person involved in a trial, whether they are a participant, health professional or researcher, is influenced by their own beliefs, attitudes and experiences which consciously or unconsciously affect the way in which they engage with the research process. This creates cultural expectations, both positive and negative, which affect how other people engage with the trial. This study found that organisational culture and underlying assumptions were rarely acknowledged and that they only become apparent if they were challenged in some way. Furthermore since those who determined the cultural norms and ethos of the research environment were ambivalent about the trial there was insufficient commitment to support the considerable energy, time and resource demands required for the trial to be a success. The disparity in influence that members of different healthcare professions exert has been well documented [23,24] and here, the effects of this were seen beyond healthcare delivery and into how research agendas are set and enacted.

### Strengths and limitations

Our sampling of trial participants and staff involved in the delivery of the interventions have allowed both perspectives to be explored. Similarly, although we acknowledge that our sample of staff is relatively small, we have included accounts of both staff directly involved in the trial and those only indirectly involved. In addition the challenges faced by both participants and healthcare staff that were identified in these interviews appear to have 'relevance' [25] to trials of complex healthcare interventions beyond this particular trial in this particular setting. A limitation of the study is the lack of a voice for those who declined to take part in the trial itself. For ethical and practical reasons we were not able to approach any individual who had already declined to take part in the trial. Another limitation of this study is that the interviews were conducted by LB who was closely associated with the trial by both patients and staff. This may have constrained interviewees' views on either the intervention(s) or the trial.

### Transferability

Many of the issues in this paper relate to a particular intervention, the way it was evaluated and the environment in which this took place. Despite this, many of the issues discussed are pertinent to the evaluation of complex health service interventions more generally. By definition complex interventions are difficult to standardise, blind and regulate. Furthermore they are far more likely to be developed as a result of the determination and dedication of clinical staff, and need to be tested within the competing pressures of the clinical environment.

## Conclusions

A lack of scientific evidence as to the efficacy of an intervention does not preclude staff and patients holding strong views about the benefits of an intervention. The evaluation of complex interventions should, where possible, facilitate not restrict that complexity. Although MRC guidance on evaluating complex interventions [3] provides a useful framework for the successful conduct of trials of this sort, the evolution of interventions in healthcare and their subsequent evaluation does not always proceed in clearly defined stages. Within this more fluid process, two elements appear crucial. First, pilot studies should test the process of recruitment and randomisation, examining the views and experiences of staff and participants on this element of the research process. The reality of randomising a service is often far more difficult for healthcare providers than they anticipate. A more formal pilot study is unlikely to be conducted without funding, but it has the advantage of greater separation between the design and testing of the intervention. Secondly, an understanding of the way a particular service operates is not enough, an insight into the organisational culture is necessary. Findings from this study strongly suggest that within the local environment where the trial is conducted, acquiescence from those in positions of authority is insufficient; commitment to the trial is required.

## Competing interests

The authors declare that they have no competing interests.

## Authors' contributions

LB, AA and KC conceived and designed the study. LB conducted the research interviews. LB and AA analysed the data. All authors helped to draft the final manuscript.

## Pre-publication history

The pre-publication history for this paper can be accessed here:

http://www.biomedcentral.com/1471-2288/11/24/prepub
